# Diagnostic Performance of the Tear Meniscus Osmolarity Measurement for Dry Eye Disease in Rheumatoid Arthritis Patients

**DOI:** 10.3390/diagnostics13182994

**Published:** 2023-09-19

**Authors:** Paola De La Parra-Colin, Karen Palacios-Aguirre, Andrea Perez-Pria-Kasusky, Rolando Espinosa-Morales, Alberto Hidalgo-Bravo

**Affiliations:** 1 Cornea and Ocular Surface Clinic, Department of Ophthalmology, Instituto Nacional de Rehabilitación Luis Guillermo Ibarra Ibarra, Ministry of Health, Mexico City 14389, Mexico; karenpalacios10@gmail.com (K.P.-A.); kzk_appk@hotmail.com (A.P.-P.-K.); 2 Department of Rheumatology, Instituto Nacional de Rehabilitación Luis Guillermo Ibarra Ibarra, Ministry of Health, Mexico City 14389, Mexico; rolespi@yahoo.com; 3 Department of Genetics, Instituto Nacional de Rehabilitación Luis Guillermo Ibarra Ibarra, Ministry of Health, Mexico City 14389, Mexico

**Keywords:** tear osmolarity, dry eye disease, rheumatoid arthritis

## Abstract

Background: The aim of our study was to evaluate the diagnostic capacity of the tear meniscus osmolarity measurement for dry eye disease (DED) in patients with rheumatoid arthritis (RA), using a portable osmometer based on electrical impedance and an integrated circuit technology (TearLab^®^ (Escondido, CA, USA)). Methods: We included 101 RA patients, 81 patients with DED and 20 without DED (controls). We measured tear osmolarity and assessed other clinical diagnostic tests as suggested by the TFOS DEWS II composite reference standard diagnostic criteria for DED using Ocular Surface Disease Index (OSDI), Five-item Dry Eye Questionnaire (DEQ-5), fluorescein tear break-up time (F-TUBT), ocular surface staining (SICCA score), and other clinical parameters to classify DED subtypes. We analyzed the agreement between osmolarity and the TFOS DEWS II composite reference standard for DED diagnosis. We conducted receiver operating characteristic (ROC) curve analyses using the DED variable and its subtypes as dependent variables and the continuous variable for osmolarity or the inter-eye difference in osmolarity as independent variable. Sensitivity, specificity, and area under the curve for all potential cut-off points were obtained and reported from ROC curves. Results: We found that tear meniscus osmolarity had a low diagnostic capacity for DED (AUC = 0.57). Tear meniscus osmolarity measurement had a sensitivity of 35% and a specificity of 80% with a kappa level of agreement of 0.08 compared to the TFOS DEWS II composite reference standard. The low diagnostic capacity of the tear meniscus osmolarity was similar for aqueous-deficient DED and for evaporative DED, being only fair for severe DED with a 57% sensitivity and 80% specificity and a kappa level of agreement of 0.36. Conclusions: Our findings suggest that in patients with RA, tear meniscus osmolarity measured by the TearLab^®^ showed low sensitivity, low specificity, and limited agreement with the TFOS DEWS II composite reference standard for DED diagnosis.

## 1. Introduction

Rheumatoid arthritis (RA) is one of the most prevalent chronic autoimmune diseases globally [1]; it affects synovial tissues, but several extraarticular manifestations can also be present. In the eye, these manifestations can include dry eye disease (DED), episcleritis, scleritis, ulcerative peripheral keratitis, among others [2]. Dry eye disease is the most frequent ocular manifestation in RA patients, generally associated with secondary Sjögren’s syndrome (sSS), affecting up to 30% of patients [3]. DED can significantly impair quality of life in affected patients particularly in clinically severe disease or in symptomatic patients [4]. DED can impact visual function, general health status, and productivity [5]; also, as disease severity increases, the perceived quality of life is worse, especially in relation to mental well-being [6]. According to the last international consensus on dry eye disease from the Tear Film and Ocular Surface Society (TFOS) Second Dry Eye Workshop (DEWS II), DED is defined as a multifactorial disease of the ocular surface characterized by a loss of homeostasis of the tear film, and accompanied by ocular symptoms, in which tear film instability and hyperosmolarity, ocular surface inflammation and damage, and neurosensory abnormalities play etiological roles [7].

One of the historical limitations for the diagnosis of DED has been the lack of a “gold standard” diagnostic test; therefore, the TFOS DEWS II international consensus proposed a set of clinical tests, i.e., a composite reference standard for the diagnosis and classification of DED [4,8]. The composite reference standard [8] to diagnose DED should include the following: (a) a positive screening test, either DEQ-5 (Five-item Dry Eye Questionnaire) or OSDI (Ocular Surface Disease Index) questionnaires, and b) at least one positive altered homeostasis marker, either, decreased tear break-up time (TBUT), tear film osmolarity ≥ 308 mOsm/L in either eye or an inter-eye difference of >8 mOsm/L, or ocular surface staining with fluorescein for the corneal epithelium or lissamine green for the conjunctival epithelium.

Tears are isotonic as they are secreted by the lacrimal glands; physiologically, tear film increases its osmolarity as we are awake due to evaporation. Pathological factors that can play a role in tear hyperosmolarity are an increased area of ocular surface exposure, or a decreased blinking frequency. Also, inadequate characteristics in the quality or quantity of the tear film lipid layer can increase evaporation of the tear film [9]. But the most severe form of tear film hyperosmolarity is a decreased production of tears due to direct damage to the lacrimal glands, as in Sjögren’s syndrome [10]. Tear film hyperosmolarity causes damage to the ocular surface epithelium and activates an inflammatory cascade [11]. The endpoint of such events is atrophy and dead epithelial cells, goblet cell loss, more inflammation, and damage to the ocular surface [12,13].

Although tear film hyperosmolarity is a well-known biomarker of DED, measuring precorneal tear film osmolarity has been a difficult task because of problems in tear film collection and the need of high-end laboratories for analytical procedures. Previous techniques for tear osmolarity measurements have been the freezing point depression method and the vapor pressure method; these are time consuming and require technical expertise. In 2008, a new device for measuring tear osmolarity was developed, the TearLab Nanoliter Osmometer (TearLab, Escondido, CA, USA). This is a point-of-care device that measures the osmolarity of the tear meniscus by electrical impedance and an integrated circuit technology that needs a relatively small amount of tear sample (~50 nL) [14]. Several studies have been published concluding that TearLab osmolarity measurement is the best test for DED diagnosis, useful to classify DED severity and to evaluate treatment outcomes better than other clinical parameters [15,16,17]. Nevertheless, other studies have shown that tear osmolarity measured by the TearLab has a low sensitivity and low specificity to discriminate between healthy and DED affected individuals [18,19,20,21]. Potential explanations for these mixed results are varied, but could include the estimation of sensitivity and specificity of osmolarity when osmolarity is already included as a criterion for DED diagnosis; the type of patient included (mild vs. normal, severe vs. normal); and even the funding source of the study, i.e., studies funded by TearLab producers, tend to show better results than independent studies [17].

Some of the reasons to explain the low sensitivity of the TearLab measurements are the high variability in the osmolarity values and the controversy in determining the best cut-off point to discriminate among no-DED, mild, moderate, and severe DED. Cut-off values to discriminate between DED and non-DED individuals have varied a lot in the literature, with a range from 294 to 316 mOsm/L [22]. Furthermore, it has been shown that as DED severity increases, the variability in osmolarity between eyes also increases [23]. This provides a different source of information to detect DED, beyond the raw measurement of osmolarity of each eye. Currently, a difference of 8 mOsm/L between eyes is also considered to be a sufficient criterion to diagnose DED, regardless of the absolute osmolarity value [4,17].

Diagnosing DED is rather complex since it is based on several clinical tests that can be variable, inexact, and highly dependent on the examiner clinical experience. We aimed to evaluate the performance of the TearLab osmolarity in patients with RA, since the prevalence of sSS in RA patients can be as high as 30%, which is an aqueous-deficient or mixed DED subtype [3]; also, RA affects predominantly women in the fourth or fifth decade of life, when evaporative dry eye disease becomes more frequent [24]. Thus, RA patients provide a wide variety of DED presentations and severities to explore the validity of the TearLab device. For this purpose, from a pool of RA patients, we identified those that complied with TFOS DEWS II criteria (DED) from those who did not (no-DED, controls) and compared osmolarity and inter-eye osmolarity differences between them. We also estimated ROC curves to assess diagnostic performance across osmolarity cut-off points for DED overall and by DED subtype (aqueous-deficient, evaporative, mixed, and severe).

## 2. Materials and Methods

We conducted a cross-sectional study in the Cornea and Ocular Surface Clinic in our institution to evaluate a consecutive group of patients with RA who had been diagnosed, according to the ACR/EULAR 2010 diagnostic criteria [25], in the Rheumatology Department at a tertiary health care institution. This study adhered to the Helsinki Declaration tenants and was approved by our research institutional board with approval number 37/18. All participants were provided information about the objective and procedures of the study, agreed to participate, and signed a written informed consent.

One hundred and one patients were included in the study from September 2018 to January 2019; exclusion criteria were anophthalmia or ptisis bulbi, asymmetrical structural and/or functional anomalies of the eyelids, any acute ocular inflammatory condition other than DED, and patients who were unable to suspend topical ocular medications (except for preservative-free lubricant) or contact lens wear, at least, one week before the evaluation.

### 2.1. Instrumentation and Procedures

All patients included in the study were evaluated by the same senior ophthalmologist, an expert in cornea and ocular surface diseases. The clinical evaluation always took place in the afternoon between 3 p.m. and 6 p.m., in the same office. Only four patients were evaluated each day. Patients were asked to suspend all eyedrops one week before the evaluation, except for preservative-free lubricants. Lubricants were allowed until 3 h before clinical examination.

On evaluation day, patients were asked to respond to both OSDI and DEQ-5 questionnaires. Also, questions about their over-all health, systemic medications, and clinical history were asked. All procedures for ocular surface examination were performed in the right eye first followed by the left eye in the following order:

1. **Tear meniscus osmolarity measurement** was performed with the TearLab osmometer (TearLab, Escondido, CA, USA). The osmometer was calibrated every day before evaluations according to the manufacturer instructions to ensure optimal performance. We could not obtain valid measures for three patients since their test cards timed out twice; we hypothesize that this could be due to low tear volume, as this occurs in severe predominantly aqueous-deficient DED cases. Thus, those measurements were missing and excluded from all osmolarity-related analyses.
2. **Schirmer’s test without anesthesia** was performed by inserting filter paper strips (TearFlo, HUB Pharmaceuticals, LLC., Scottsdale, AZ, USA) in the external portion of the lower lid of both eyes, maintaining lid closure, for 5 min.
3. **Ocular surface biomicroscopy** was performed under the same slit lamp (CSO SL-9900 Elite, Fontaniva, Italia), and care was taken not to manipulate the eyelids nor to use over illumination.
4. **Tear meniscus height** was measured with the slit lamp beam marker.
5. **Conjunctival hyperemia** was graded according to Ephron’s classification [26].
6. **Conjunctival staining** was graded using lissamine green ophthalmic strips (Green-Glo, HUB Pharmaceuticals, LLC.Rancho Cucamonga, CA, USA). The strip was wetted with balanced salt solution and inserted in the inferior cul-de-sac at the external portion of the lower lid; one minute after being applied the grading was performed according to the SICCA score [27]. Also, any staining of the conjunctival epithelium in the posterior lid margin was registered.
7. **Fluorescein tear break-up time** (F-TBUT) was performed with fluorescein sodium ophthalmic strips (Bio-Glo, HUB Pharmaceuticals, LLC., Rancho Cucamonga, CA, USA). The strip was wetted with balanced salt solution and inserted in the inferior cul-de-sac at the external portion of the lower lid. Patient was instructed to blink three times and the measurement was performed under cobalt blue light and a yellow filter to increase contrast.
8. **Fluorescein corneal epithelial staining**. Right after F-TBUT was evaluated, we graded corneal epithelial staining according to the SICCA ocular surface staining score [27].
9. **Meibomian gland expressibility and meibum evaluation** was classified according to Bron’s grading of lid changes [28].

### 2.2. Criteria for DED Diagnosis and Classification

Patients were classified as having DED or not (controls) according to the TFOS DEWS II composite reference standard. Osmolarity was excluded from the standard since its inclusion would have introduced autocorrelation. DED and its classification were integrated within groups as follows:

1. **Controls**: patients that did not meet the DED diagnosis criteria.
2. **Dry eye disease**: OSDI >12 points or DEQ-5 > 5 points and a positive clinical sign: (a) F-TBUT < 5 s and (b) ocular surface staining with a SICCA score > 2.
3. **Aqueous-deficient dry eye disease**: DED criteria plus having a lacrimal meniscus height ≤ 0.2 mm and a Schirmer’s test without anesthesia < 5 mm.
4. **Evaporative dry eye disease**: DED criteria plus having < 5 expressible meibomian glands in the medial third of the lower eyelid and/or meibum with opacity and increased viscosity.
5. **Mixed dry eye disease**: Having criteria for both aqueous-deficient and evaporative dry eye disease.
6. **Severe dry eye disease**: DED criteria plus F-TBUT < 1 s and ocular surface staining with a SICCA score > 6.

### 2.3. Statistical Analysis

We estimated the sample size to detect a difference in osmolarity equal to the difference observed by Jacobi et al. [16]; they reported a median of 320 mOsm/L in participants with moderate-to-severe DED (IQR 301–324 mOsm/L) and 301 mOsm/L in participants without DED (IQR 298–304 mOsm/L). Using this information, we approximated a normal distribution to estimate the expected mean and standard deviation being 301 mOsm/L (SD 4.54) for patients without DED and 314.7 mOsm/L (SD 17.24) for patients with DED [29,30]. Using this approximation and assuming a level of significance of 0.05 and power of 0.8, we estimated a minimum of 15 patients on each group (DED and non-DED).

For descriptive analysis, we estimated frequencies and percentages for categorical variables, mean and standard deviation for continuous variables with normal distribution, and median and interquartile range for numerical variables with non-normal distributions. We assessed differences in sociodemographic and clinical characteristics by DED status using median tests for non-normal numerical variables, *t*-test for normal numerical variables, and Chi-square tests for categorical variables.

According to the literature, tear meniscus osmolarity of 308 mOsm/L and higher has been used as the cut-off point to classify patients as having DED [4]. As a first step, we analyzed the agreement between tear meniscus osmolarity (<308 mOsm/L or ≥308 mOsm/L) and other individual clinical parameters used to identify DED: OSDI, DEQ-5, F-TBUT, SICCA score, and Schirmer’s test. For agreement, we calculated Kappa coefficient, sensitivity, and specificity. Then, we analyzed the agreement between osmolarity and the TFOS DEWS II composite reference standard to diagnose DED, excluding tear meniscus osmolarity to avoid inducing correlation by including this variable in the DED diagnosis. Also, since an alternative criteria to identify DED is the inter-eye difference in tear meniscus osmolarity, >8 mOsm/L regardless of the absolute value, [4] we stratified patients by DED status (no DED, DED, and severe DED) and calculated the absolute inter-eye difference in osmolarity to assess the differences in the distribution across DED groups. These clinimetric parameters were calculated for the osmolarity results of the right eye first, to avoid the potential dilution effect of tear reflex that could potentially occur in the left eye, and then for the osmolarity result of either eye or the inter-eye difference in osmolarity.

To evaluate other potential cut-off points for osmolarity and osmolarity inter-eye difference, we conducted receiver operating characteristic (ROC) curve analyses using the DED variable and its subtypes compared to non-DED patients (controls) as dependent variables and the continuous variable of tear meniscus osmolarity, or the inter-eye difference in osmolarity, as independent variable. Sensitivity, specificity, and the area under the curve for all potential cut-off points were obtained and reported from ROC curves. All statistical analyses were conducted in Stata 14.0 (College Station, TX, USA).

## 3. Results

### 3.1. Clinical and Demographic Characteristics

Table 1 shows demographic and clinical characteristics of participants. We evaluated 101 patients with confirmed RA. We found 81 patients with DED (80.2%) and 20 patients without DED (19.8%). As expected, we observed a higher frequency of women (85.1%) than men (14.9%), and these frequencies were similar between patients with or without DED. Mean age was 56.7±11.7 years and was significantly higher in patients with DED (58.2 ± 11) than without DED (50.6 ± 12.8) (*p* = 0.008).

Only right eye clinical tests results for DED are presented (Table 1) since this was the first eye evaluated in all cases and to reduce bias in the osmolarity measurement due to the reflex lacrimal stimulus that could have happened in the second eye after the first eye was measured. We found abnormal values for almost every DED clinical test proposed by the TFOS DEWS II, in patients with DED except for tear meniscus osmolarity. TearLab osmolarity difference was not statistically significant, with a median of 303 mOsm/L (RQ 291, 313) in DED patients vs. 295 mOsm/L (RQ 286, 304) in patients without DED (*p* = 0.059).

Other clinical parameters that we evaluated were Schirmer’s test without anesthesia with a median of 5 mm (IQR 3, 11) in patients with DED and 12 mm (IQR 7, 29) in patients without DED (*p* = 0.002). Difference in lacrimal meniscus height was not significant between patients with and without DED, but we did find a difference in the grade of bulbar conjunctival hyperemia that was higher in patients with DED (*p* = 0.007). Also, the number of expressible meibomian gland orifices was different between groups (*p* = 0.023); however, we found no difference in the meibum characteristics in patients with or without DED.

### 3.2. DED Prevalence

The prevalence of DED was 80.2%; among these, the subtype prevalence was 41.6% for evaporative DED, 13.9% for aqueous-deficient DED, and 24.8% for mixed DED (Table 2).

### 3.3. Discriminative Capacity of Tear Meniscus Osmolarity in DED

Diagnostic performance indicators of tear meniscus osmolarity using the proposed cut-off point of 308 mOsm/L to detect DED, DED subtypes, and other diagnostic tests are shown in Table 3. We show the results for the right eye and for either eye or for the inter-eye difference in osmolarity. TearLab osmolarity to diagnose DED had a 35% sensitivity and 80% specificity. Kappa level of agreement were low, with kappa values below 0.4 in all cases.

**Figure 1:**
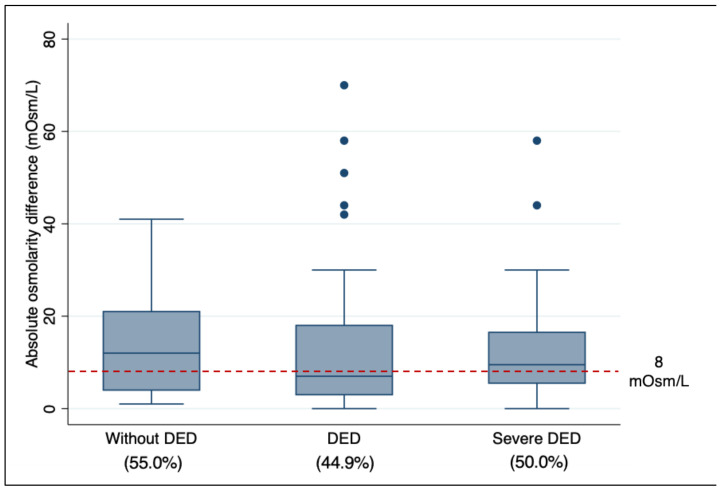
Absolute inter-eye osmolarity difference in mOsm/L comparing patients without DED, with DED, and with severe DED. Red dashed line shows the proposed cut-off inter-eye difference of 8 mOsm/L to differentiate between DED and non-DED patients. Percent under labels are the proportion of patients with an inter-eye difference >8 mOsm/L in each group.

In Figure 1, we show the absolute inter-eye tear meniscus osmolarity difference between groups. In patients without DED, we found a median of 12 mOsm/L (IQR 4, 21), in patients with DED a median of 7 mOsm/L (IQR 3, 18), and in patients with severe DED a median of 9.5 (IQR 5.5, 16.5). For the inter-eye difference of >8 mOsm/L, we found this difference with a frequency of 55% for patients without DED, 44.9% for patients with DED, and 50% for those with severe DED. We found an average inter-eye difference in osmolarity of 4.42 mOsm/L for all participants and of 5.6 mOsm/L in patients with DED. In average, tear meniscus osmolarity for the right eye was higher than for the left eye, 301.2 vs. 296.8 mOsm/L, respectively.

**Figure 2:**
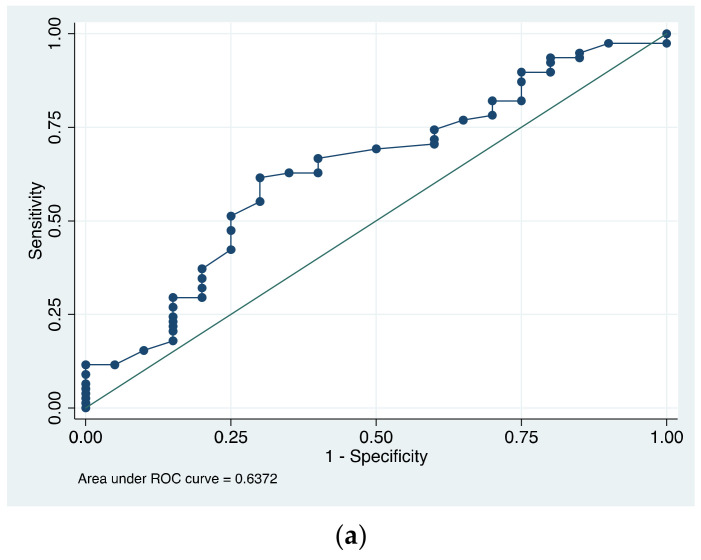

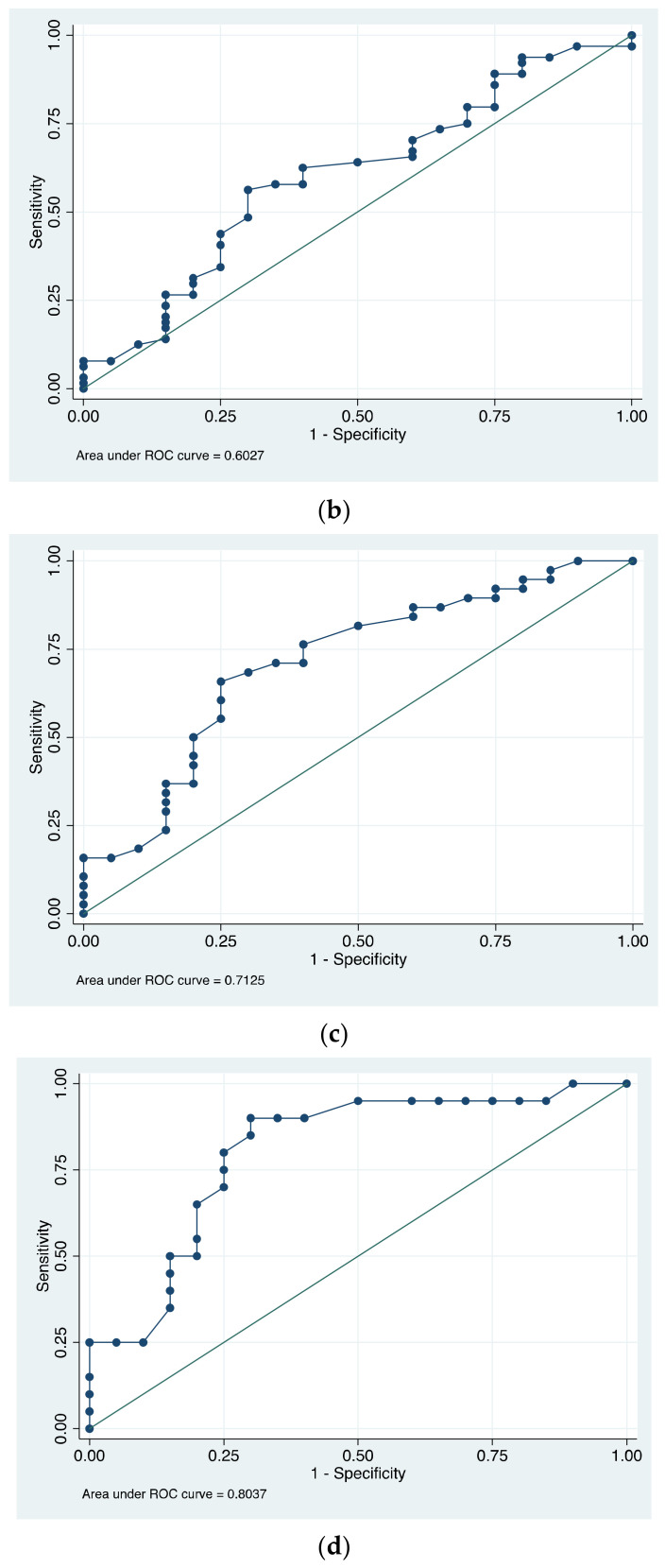
ROC curves that show the discriminative capacity of tear meniscus osmolarity for DED and DED subtypes. (**a**) The area under the curve (AUC) was low for DED and for each subtype; (**b**) evaporative DED and (**c**) aqueous-deficient DED; (**d**) being only acceptable for severe DED.

**Figure 3:**
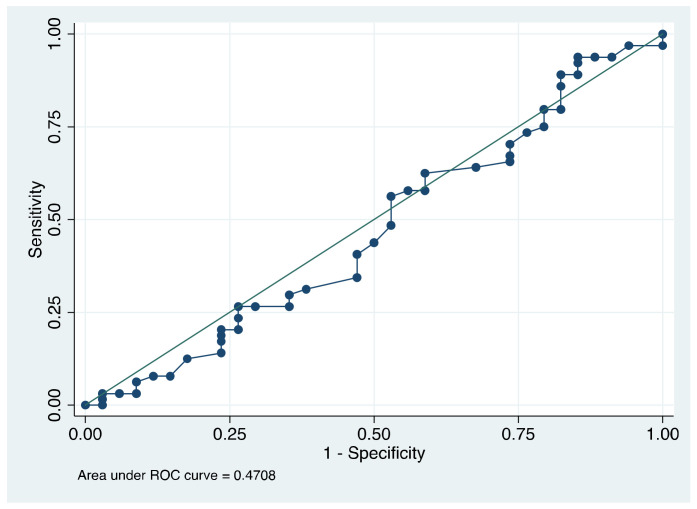
ROC curve that shows the discriminative capacity of the tear meniscus osmolarity inter-eye difference for DED. The area under the curve (AUC) was lower than 0.5.

Finally, to evaluate the discriminative capacity of TearLab osmolarity in patients with and without DED, we used ROC curves (Figure 2). The area under the curve (AUC) was low in each subtype of DED, except for severe DED: DED (AUC = 0.637), evaporative DED (AUC = 0.602), aqueous-deficient DED (AUC = 0.712), and severe DED (AUC = 0.803). For the discriminative capacity of the inter-eye difference of >8 mOsm/L, regardless of the tear meniscus osmolarity absolute value, between patients with and without DED, the area under the curve was the lowest (AUC = 0.47) (Figure 3).

## 4. Discussion

The aim of our study was to evaluate the ability of the tear meniscus osmolarity to identify DED in patients with rheumatoid arthritis, using a point-of-care osmometer based on electrical impedance and an integrated circuit technology (TearLab^®^). We found that tear meniscus osmolarity had a low diagnostic capacity for DED (AUC = 0.57). In patients with rheumatoid arthritis, tear meniscus osmolarity measurement had a sensitivity of 35 percent and a specificity of 80 percent with a kappa level of agreement of 0.08 compared to the composite reference standard for DED diagnosis, excluding osmolarity, as proposed by the TFOS DEWS II. The low diagnostic capacity of the tear meniscus osmolarity was similar for aqueous-deficient DED and for evaporative DED, being only fair for severe DED with a 57% sensitivity and 75% specificity and a kappa level of agreement of 0.29. Furthermore, we found that TearLab osmolarity had a limited agreement with other clinical parameters used to diagnose DED.

Tear osmolarity has been considered as a biomarker for DED. The TFOS DEWS II established a cut-off point ≥308 mOsm/L in either eye or an inter-eye difference >8 mOsm/L, regardless of the absolute measured value, to diagnose DED [4]. However, other studies have challenged the diagnostic capacity of this test, arguing a great variability and overlapping in the measured values even in people without DED [20,21]. In our study, we found a low discriminative capacity when comparing the TFOS DEWS II composite reference standard for DED diagnosis and tear meniscus osmolarity. The low sensitivity that we found is comparable to other studies: 67% with a cut-off value of 294 mOsml/L for moderate DED in university students [31] and 40% with a cut-off value of 310 mOsml/L for DED associated with sSS [32]. These authors also reported a poor diagnostic performance for the TearLab. In 2018 [33], a systematic review that included 33 studies evaluated the TearLab osmometer and found large variability in osmolarity and showed that if 308 mOsm/L was used as cut-off point, 25% of healthy people would have been diagnosed with DED. Few studies have reported tear osmolarity in RA patients. Craig et al. in 1995 reported a significantly increased tear osmolarity using the freezing point depression method compared to non-RA participants [34]; their results are not comparable to ours since the osmolarity they measured was from the corneal tear film not from the tear meniscus. We could not find other studies using the TearLab to assess diagnostic ability in RA patients. Schargus et al. found a correlation between higher levels of tear meniscus osmolarity (≥316 mOsm/L) and disease activity through the DAS-28 ESR score in RA patients, but they did not assess the sensitivity and specificity of the TearLab osmometer for DED diagnosis [35].

The criteria to use a cut-off point of 308 mOsml/L to establish a diagnosis of DED was proposed by Lemp et al., in 2011 [15], by means of a multicenter study (10 centers in EU and USA) that included 298 consecutive participants between the ages of 18 and 82 years, with a 2:1 ratio of DED suspects to healthy participants. According to their analysis, the 308 mOsm/L cut-off value maximized the percent of patients correctly classified, yet no sensitivity/specificity values were provided. Interestingly, the report does not provide information on the clinical criteria used to identify DED suspects and healthy subjects, despite using multicenter design that required standardization of DED definition. Furthermore, they used a post hoc expert consensus panel to define cut-off points for different clinical indicators, which were substantially different to the TFOS DEWS II composite reference standard used in our study to diagnose DED. Also, we assessed through ROC curves all possible cut-off points for sensitivity and specificity, avoiding any expert opinion. The cut-off point that maximized the discriminative capacity varied according to DED subtype: 300 mOsm/L for global DED, 302 mOsm/L for aqueous-deficient DED, and 300 mOsm/L for evaporative DED. The sensitivity was always below 61% and the area under the curve below 0.70. For severe DED, the best cut-off point was 302.5 mOsm/L, producing a sensitivity of 90% and a specificity of 70% (AUC = 0.80). These findings suggests that the diagnostic capacity of TearLab in RA is limited for any of the characterizations of DED, except for severe DED. However, the added information provided by TearLab may be irrelevant in severe DED cases in RA patients, since clinical manifestations tend to be salient enough to establish the diagnosis.

Besides having compared the discriminative capacity of the TearLab osmolarity against the TFOS DEWS II composite reference standard for DED diagnosis, we also evaluated the diagnostic performance of the TearLab osmolarity against each clinical diagnostic parameter independently. Overall, osmolarity had a poor level of agreement with the other clinical parameters. Even though we did not find other studies reporting kappa level of agreement with TearLab osmolarity, other authors have reported Spearman correlation coefficients with comparable results. Alves et al. [32] reported a limited agreement with OSDI (0.07), F-TBUT (0.09), fluorescein staining (0.07), and Schirmer’s test (0.04). Similar results were found by Caffery et al. [36] where TearLab osmolarity had no significant correlation with DEQ-5 scores in subjects with average osmolarity (correlation coefficient 0.02), nor in patients with high osmolarity (correlation coefficient 0.03). These results suggest that the deficient diagnostic performance of the osmolarity test is not restricted to the composite reference standard for DED diagnosis taken as a whole, but also to each parameter independently.

In our study, the TearLab osmolarity test showed a high variability within patients, regardless of their clinical condition, either non-DED, DED or severe DED. This finding has also been reported by others [20,33], with different explanations, and that the TearLab osmometer is only determes tear meniscus osmolarity and not the osmolarity of the precorneal tear film itself, which can increase variability since tear meniscus osmolarity changes are more challenging to measure, and also due to stimulation of reflex tear secretion. We measured the osmolarity in the right eye first followed by the left eye, which allowed us to estimate differences attributable to the measurement order. We found that, on an average, the right eye had higher osmolarity values than the left eye (301.2 vs. 296.8 mOsm/L) with an average difference of 4.42 mOsm/L; this suggests that the TearLab osmolarity test might induce reflex tear secretion that reduces the osmolarity of the tear meniscus being more evident when measuring the second eye. An absolute inter-eye osmolarity difference of 8 mOsm/L or higher has been proposed as a diagnostic criterion for DED. However, the median absolute inter-eye difference was higher in patients without DED than with DED; and we found a similar proportion of patients with > 8 mOsm/L of inter-eye difference across groups without DED, with DED, and with severe DED (Figure 1). Moreover, as shown in Figure 3, the discriminative capacity of this parameter was very low (AUC = 0.43). Our findings suggest that the inter-eye osmolarity difference may not be informative to diagnose DED.

In our study, we found a prevalence of 80.2% for DED. Although this prevalence is high, other authors have reported similar findings. In a cohort of 70 patients with RA, Brun et al. found a prevalence of eye dryness symptoms in 80% [37]. Another prospective study in patients with RA reported a prevalence of secondary Sjögren’s syndrome (sSS) of 22.2% and 46.7% with DED [38]. Fujita et al. [39] found in a cohort of patients with RA a prevalence of sSS of 10% and a prevalence of 90% for DED suspects. These findings can be explained by the fact that clinical manifestations of rheumatoid arthritis occur on average between the fourth and fifth decade of life and affect more frequently women than men [24]. Around this age, predominantly evaporative DED can also be more prevalent in women due to hormonal changes, specifically low androgen levels that change the meibum composition rendering instability of the precorneal tear film [40,41]. Also, the prevalence of sSS in RA patients can be as high as 30% [3], which will be a predominantly aqueous-deficient or mixed DED, and can be very severe. In our study, the prevalence of aqueous-deficient DED was 13.9% and 23.4% for mixed DED. This data shows the importance of an adequate and timely diagnosis of DED in the RA population.

Our study has some limitations that need to be discussed. Our study is restricted to dry eye in RA patients and as such our results can only be generalized to this type of patients. The low agreement that we found between TearLab osmolarity and other clinical diagnostic parameters for DED could be attributed to the homogeneity of these parameters in RA patients; however, 20 participants were classified as non-DED patients, which allowed us to have a large enough control group according to our estimated sample size. Nevertheless, for some comparisons, particularly when splitting the sample in DED subtypes (aqueous-deficient, evaporative, and mixed), statistical power could be insufficient. We were unable to perform some measurements that depend on more sophisticated diagnostic equipment such as non-invasive TBUT, non-invasive meniscometry, or tear film interferometry to be more precise about the classification of DED subcategories; however, our study aimed to assess osmolarity under regular diagnostic conditions to reflect its usefulness in everyday practice. Tear reflex is a potential confounder in the evaluation of tear osmolarity; to reduce this possibility, we conducted the osmolarity measurement in the right eye first, followed by the left eye. Through this procedure, we minimized the potential dilution effect of tear reflex in the right eye, allowing us to estimate the impact of the reflex in the comparison between the right and left eyes.

## 5. Conclusions

In conclusion, our study suggests that tear meniscus osmolarity measured by the TearLab osmometer is non-informative to differentiate DED from non-DED in RA patients. TearLab osmolarity showed a low sensitivity and limited agreement with the TFOS DEWS II composite reference standard for DED diagnosis, as well as with individual indicators of DED as SICCA ocular staining score or Schirmer’s test. TearLab diagnostic capacity should be analyzed by other research groups with no conflicts of interest to either corroborate or refute our findings. The diagnostic advantage of TearLab needs to be clearly justified as the relative high cost of this platform for clinicians or health institutions, particularly in middle- and low-income countries, and implies a significant investment. For the time being, we recommend to cautiously consider the use of the TearLab osmometer as a diagnostic tool for DED in patients with RA.

## Figures and Tables

**Table 1:** Clinical and demographic characteristics of patients with rheumatoid arthritis.

	All (*n* = 101)		With DED (*n* = 81)		Without DED (*n* = 20)		
Variable	%/SD/IQR		%/SD/IQR		%/SD/IQR		*p*-Value
**Sex**							
*Women (n, %)*	86	85.1	69	85.2	17	85.0	0.610
*Men (n, %)*	15	14.9	12	14.8	3	15.0	
**Age** (mean, SD)	56.7	11.7	58.2	11.0	50.6	12.8	0.008
**Time from RA diagnosis** (median, IRQ)	12	(6, 17)	12	(7, 18)	7	(3, 14)	0.029
**OSDI** (mean, SD)	27.8	20.9	30.9	19.9	15.2	20.6	0.002
**DEQ-5** (median, IQR)	7	(3, 11)	8	(4, 11)	4.0	(1, 8)	0.002
***TearLab^®^* osmolarity** (mean, SD)	301.2	(16.1)	302.8	(16.2)	295.2	(14.7)	0.059
**Schirmer’s test** (median, IQR)	6	(3, 14)	5	(3, 11)	12	(7, 29)	0.002
**F-TBUT** (median, IQR)	0	(0, 3)	0	(0, 2)	3	(1, 7)	<0.001
**SICCA-score** (median, IQR)	3	(0, 6)	3	(0, 7)	0	(0, 2)	<0.001
**Lacrimal meniscus height**							
*<0.3 mm (n, %)*	44	43.6	34	42.0	10	50.0	0.331
*0.3 mm (n, %)*	34	33.7	30	37.0	4	20.0	
*>0.3 mm (n, %)*	23	22.8	17	21.0	6	30.0	
**Conjunctival hyperemia** *Efron’s grading scale*							
*0-normal (n, %)*	4	4.0	2	2.5	2	10.0	0.007
*1-trace (n, %)*	16	15.8	8	9.9	8	40.0	
*2-mild (n, %)*	38	37.6	33	40.7	5	25.0	
*3-moderate (n, %)*	36	35.6	31	38.3	5	25.0	
*4-severe (n, %)*	7	6.9	7	8.6	0	0.0	
**Expressible Meibomian glands**							
*<5/10 (n, %)*	42	41.6	29	35.8	13	65.0	0.023
*≥6/10 (n, %)*	59	58.4	52	64.2	7	35.0	
**Meibum characteristics**							
*Transparent and normal viscosity (n, %)*	9	8.9	6	8.1	3	16.7	0.293
*Opaque and increased viscosity (n, %)*	57	56.4	45	60.8	12	66.6	
*“Toothpaste” viscosity (n, %)*	26	25.7	23	31.1	3	16.7	

RA: rheumatoid arthritis, OSDI: Ocular Surface Disease Index, DEQ-5: Five-item Dry Eye Questionnaire, F-TBUT: fluoresceine tear break-up time, SICCA-score: SICCA ocular staining score.

**Table 2:** Prevalence of DED and DED subtypes.

DED and DED Subtypes	*n* (%)
**DED**	
*No*	20 (19.8)
*Yes*	81 (80.2)
**Evaporative DED**	
*No*	59 (58.4)
*Yes*	42 (41.6)
**Aqueous-deficient DED**	
*No*	87 (86.1)
*Yes*	14 (13.9)
**Mixed DED**	
*No*	76 (75.2)
*Yes*	25 (24.8)

DED: dry eye disease.

**Table 3:** Sensitivity, specificity, agreement, and area under the curve of TearLab^®^ osmolarity and DED diagnostic parameters and TFOS DEWSII composite reference standard for DED diagnosis.

	Right Eye Osmolarity (≥308 mOsm/L)				Osmolarity in Either Eye (≥308 mOsm/L) or Difference in Osmolarity (>8 mOsm/L)			
	Sensitivity (%)	Specificity (%)	Kappa	AUC *	Sensitivity (%)	Specificity (%)	Kappa	AUC *
**Diagnostic parameters**								
*OSDI*	27	56	−0.12	0.41	55	33	−0.10	0.44
*DEQ-5*	28	61	−0.09	0.44	60	44	0.04	0.52
*F-TBUT*	35	92	0.09	0.63	58	42	0.00	0.50
*SICCA-score*	46	84	0.29	0.65	65	49	0.14	0.57
*Schirmer’s test*	45	78	0.24	0.62	62	44	0.06	0.53
**Integrated diagnosis**								
*Dry eye disease (DED)*	35	80	0.08	0.57	58	40	−0.01	0.49
*Aqueous-deficient DED*	44	80	0.19	0.62	59	40	0.19	0.50
*Evaporative DED*	30	80	0.05	0.55	55	40	0.06	0.48
*Severe DED*	57	80	0.36	0.69	76	40	0.37	0.58

* Cut-off points for diagnostic parameters: OSDI > 12 points; DEQ5 > 5 points; F-TBUT < 5 s; SICCA > 2 points; and Schirmer’s test without anesthesia < 5 mm.

## Data Availability

The data presented in this study are openly available in FigShare at 10.6084/m9.figshare.24079392 (accessed on 3 September 2023).
